# Prevalence of depression, anxiety and associated factors among school going adolescents in Bangladesh: Findings from a cross-sectional study

**DOI:** 10.1371/journal.pone.0247898

**Published:** 2021-04-01

**Authors:** Md. Saiful Islam, Md. Estiar Rahman, Mst. Sabrina Moonajilin, Jim van Os

**Affiliations:** 1 Department of Public Health and Informatics, Jahangirnagar University, Savar, Dhaka, Bangladesh; 2 Centre for Advanced Research Excellence in Public Health, Savar, Dhaka, Bangladesh; 3 Department of Psychiatry, UMC Utrecht Brain Center, University Medical Center Utrecht, Utrecht University, Utrecht, The Netherlands; 4 Department of Psychosis Studies, Institute of Psychiatry, Psychology & Neuroscience, King’s College London, London, United Kingdom; St. Paul’s Hospital Millenium Medical College, ETHIOPIA

## Abstract

**Background:**

Common mental disorders in early life represent a major concern as they become more complex and intense with transition into adolescence. Despite global recognition of the significance of adolescent mental health, it remains a neglected area in research and health policy in Bangladesh. This study aimed to investigate the prevalence and factors associated with depression and anxiety among school going adolescents in Bangladesh.

**Methods:**

A cross-sectional survey was conducted among 563 students aged 13–18 years at selected schools (secondary and higher secondary) in Dhaka City. After providing written informed consent, participants completed a survey examining socio-demographic variables, along with the PHQ-9 and GAD-7 scales. Logistic regression was used to examine associations between variables under examination.

**Results:**

The prevalence rates of moderate to severe levels of depression and anxiety were 26.5% and 18.1%, respectively. Based on multivariable analyses, unsatisfactory sleep (AOR = 3.17; 95% CI = 1.81–5.53, *p* < .001), cigarette smoking (AOR = 2.00; 95% CI = 1.01–3.97, *p* = .048), and anxiety (AOR = 10.47; 95% CI = 6.11–17.95, *p* < .001) were associated with depression. Anxiety was associated with being 15–16 years (AOR = 2.66; 95% CI = 1.18–6.00, p = .018), not having good perceived relationships with friends (AOR = 2.10; 95% CI = 1.24–3.56, *p* = .006) and depression (AOR = 10.22; 95% CI = 6.01–17.38, *p* < .001).

**Conclusions:**

Depression and anxiety were prevalent among school going adolescents in Bangladesh. The findings suggest epidemiological data can direct policy-level decisions regarding evaluation, prevention, and intervention of mental health conditions among school going adolescents in Bangladesh.

## Introduction

Adolescence (aged 10–19 years) can be characterized as a transitional period from childhood to adulthood. Various physical, emotional and social changes, including exposure to poverty, abuse or violence, can predispose adolescents vulnerable to mental health disorders [1]. Mental health disorders in children and adolescents have become a significant public health and mental health services concern worldwide [2]. An estimate of the World Health Organization (WHO) reported that mental disorders account for 16% of the global burden of disease and injury in people aged 10–19 years [1]. Depression and anxiety are the most common mental disorders (CMDs) among children and adolescents [3].

CMDs of adolescents have become a neglected public health problem in Bangladesh. According to WHO estimation, adolescents comprise 10.2% of the total population of Bangladesh (16.4 million; 8.4 million boys and 8.0 million girls), and suffer from suicidal behavior (i.e., suicidal ideation, suicidal ideation with a plan, attempted suicide), anxiety, loneliness, lack of close friends and substance use (i.e., tobacco, cigarette, alcohol, marijuana, multiple substances, etc.) [4].

The prevalence of mental disorders is rising among adolescents worldwide. A systematic review and meta-analysis demonstrated that the worldwide pooled prevalence of mental disorders was 13.4% in children and adolescents [5]. There have been various studies that have investigated depression and anxiety among adolescent populations in the Asian region. For example, reported prevalence rates for adolescent depression are 25.8% in India [6], 17.2% in Pakistan [3], 26.2% in Malaysia [7], 36% in Sri Lanka [8], and 52.6% in Iran [9]. Prevalence rates for adolescent anxiety are 21.4% in Pakistan [3], 28% in Sri Lanka [8], and 16.3% in Jordan [2]. These studies have opined several contributing factors to mental disorders, including social-demographic and lifestyle factors. These include sex, age, lower economic status, academic grade, father’s education, mother’s education, living with the family, alcohol intake, and satisfaction with sleep [2, 3, 7, 8].

Despite the aforementioned studies on depression and anxiety among students, there is a knowledge gap regarding depression and anxiety among adolescents in Bangladesh. A systematic review on the mental health situation of Bangladesh concluded that the overall prevalence of mental health problems ranged from 6.5–31% among adults and from 13.4–22.9% among children [10]. Although a previous study of Bangladeshi adolescents reported that the prevalence of adolescent depression was 36.6% [11], there is no study in Bangladesh that studied associated factors of anxiety and depression. Similarly, although many studies outside Bangladesh have focused on anxiety and depression, separate investigation in Bangladesh is still required, given strong cultural and sociodemographic differences between countries on the one hand and evidence of similarly strong contextual effects on common mental disorder on the other. In other words, developing public health approaches towards a mental disorder phenotype requires local inventory of prevalence as resulting from a unique mix of contextual factors. For example, the well-known sex difference in depression may not necessarily apply to Asian countries [12].

There has been no previous study investigating the prevalence of anxiety, along with depression, among school going adolescents in Bangladesh. Consequently, the present study aimed to investigate the prevalence of depression and anxiety among adolescents in Bangladesh as well as to determine factors associated with depression and anxiety. We also investigated associated factors, guided by previous research in this area.

## Materials and methods

### Participants and procedure

A cross-sectional and self-reported survey was conducted among adolescent students aged 13–18 years at selected schools (secondary and higher secondary) in Dhaka City (in Bangladesh) from November 2019 to February 2020. A multi-stage sampling technique was employed to recruit subjects for this study. In the first stage, a number of schools (primary sampling unit) were selected, conveniently located in the study area. In the selected schools, each grade is divided into several sections. Then, sections were randomly selected and students in the selected sections were then eligible to participate in the study. Overall, six schools including four secondary (grades 8–10) and two higher secondary (grades 11–12) schools, (24 classes) were included in the study. Inclusion criteria for participation included adolescent age range and willingness to enroll in the survey. Exclusion criteria were not completing the full survey.

The sample size was calculated using the following equation:

$$n=\frac{Z^{2}pq}{e^{2}}\times de;n=\frac{1.96^{2}\times .366\times (1-.366)}{.05^{2}}\times 1.5=534.9\approx 535$$

Here, *n* = number of samples; *z* = 1.96 (95% confidence level); *p* = prevalence estimate (.366); *q* = (1-*p*); e = margin of error; de = design effect for multistage sampling.

In the most recent study, Anjum et al. (2019) reported a prevalence rate of depression of 36.6% focusing on urban and semi-urban adolescents in Bangladesh [11]. For multi-stage sampling, the margin of error and the design effect are usually considered to be 5% and 1.5, respectively. In the present study, the calculated sample was to be 535. However, 563 participants were recruited to ensure adequate power for the study.

### Measures

#### Socio-demographic measures

A self-report questionnaire comprising written informed consent, questions regarding socio-demographic and lifestyle-related information, as well as psychometric scales to assess depression and anxiety, was used to collect the data. Socio-demographic data were collected, including age, sex, monthly family income, academic grade (secondary/higher secondary), relationship status (not in relationship/in a relationship), living together with family (yes/no) and perceived relationship with friends (good/not good). Socioeconomic status (SES) was categorized into three classes based on monthly family income: lower (less than 10000 Bangladeshi Taka [BDT] ≈ < 120 US$), middle (10000–20000 BDT ≈ 120–240 US$), and upper (more than 20000 BDT ≈ > 240 US$) [13, 14]. A pilot test was conducted on 30 students to confirm the reliability of the questionnaire.

#### Lifestyle-related variables

The lifestyle-related data were collected by asking questions concerning regular physical activity (yes/no), sleeping satisfaction (yes/no), number of sleeping hours per night, cigarette smoking (yes/no), and use of internet (yes/no). Sleeping hours were categorized on the basis of average daily sleeping hours as: normal (7-8h), less than average (<6h) and more than average (>9h) [13, 15].

#### Patient Health Questionnaire (PHQ-9)

The nine-item Bangla version Patient Health Questionnaire (PHQ-9) scale was used to assess depressive symptoms [16], conform previous work in Bangladesh [17–19]. This scale consists of 9 questions with a four-point Likert scale ranging from 0 *(“Not at all”*) to 3 (*“Nearly every day”*) [20]. The level of depression was categorized in five groups as minimal, mild, moderate, moderately severe, and severe based on scoring in the range of 0–4, 5–9, 10–14, 15–19, and 20–27, respectively. In the present study, those scoring in the moderate to severe range (≥10) were classed as depression positive [21]. Evidence shows that the PHQ-9 has high sensitivity (89.5%) and good specificity (78.8%) for detecting depressive symptoms among adolescents [22]. In the present study, the PHQ-9 scale was found to have good reliability (Cronbach’s alpha = .82 and mean inter-item correlation = .34).

#### Generalized Anxiety Disorder (GAD-7)

The seven-item Bangla version Generalized Anxiety Disorder (GAD-7) scale was used to assess anxiety problems among the participants, conform previous work in Bangladesh [23–25]. The scale consists of 7 items questions with a four-point Likert scale ranging from 0 (“*Not at all*”) to 3 (“*Nearly every day*”) [26]. The level of anxiety was categorized in four groups as minimal, mild, moderate, and severe based on scoring in the range of 0–4, 5–9, 10–14, and 15–21, respectively. The GAD-7 has high sensitivity (97%) and specificity (100%) for the detection of anxiety among adolescents [27]. In this study, those scoring in the moderate to severe range (≥10) were classified as having anxiety disorder [26, 27]. The GAD-7 scale was found to have good reliability (Cronbach’s alpha = .82 and mean inter-item correlation = .39).

### Statistical analysis

Data were analyzed using Microsoft Excel 2010 and SPSS Statistics version 25. Microsoft Excel was used for data entry, editing, and sorting. Finally, an excel file including all variables was imported into SPSS software. Descriptive statistics (e.g., frequencies, percentages, means, standard deviation, etc.) and first-order analysis (i.e., chi-square tests) were calculated with SPSS software. Logistic regression (both unadjusted and adjusted models) was performed, yielding odds ratios and their 95% confidence intervals, to examine associations between categorical dependent and independent variables. Analyses were univariable, yielding crude odds ratios (COR), followed by multivariable analyses with only significant factors (from univariable analyses) entered together, with the exception of anxiety and depression in the models of each other, yielding adjusted odds ratios (AOR). The association of variables was considered statistically significant if the two-sided *p*-value was less than .05.

### Ethical considerations

All procedures of the present study were performed in accordance with the Intuitional Research Ethics board. The study was approved by the Biosafety, Biosecurity, and Ethical Clearance Committee, the ethical review board of the Faculty of Biological Sciences, Jahangirnagar University, Savar, Dhaka-1342, Bangladesh. Formal permission was taken from the respective school/college authorities. Class teachers read the questionnaire and approved it to collect data prior survey. The written consent was obtained from each participant prior to data collection. The informed consent documented clearly explaining the aims and procedures of the study, risks, and benefits associated with participation, their right for voluntary participation and their right to withdraw from the study. Moreover, parents/guardians were also informed about the aim of the study through an ascent form. Participants’ information like name, mobile number, and address was not recorded, and coding was used in the questionnaires. Anonymity and confidentiality were ensured for all participants.

## Results

Participants were 55.6% male and 44.4% female, and their mean age was 15.71 years (SD = 1.48), ranging from 13 to 18 years. Descriptive statistics for all variables are presented in Table 1. The majority of respondents were not in relationship (84.9%), had upper SES (62.9%), lived together with their families (86.7%), maintained good relationships with their friends (76.4%), engaged in regular physical activities (55.4%), were satisfied with their sleep (83.3%), slept less than average (46.4%), were non-smoker (88.6%), and internet users (88.3%).

**Table 1:** Distribution of variables, and association with depression and anxiety among adolescents.

Variables	Total N = 563		Depression					Anxiety				
			*Positive*^†^		*Negative*		*p*-value	*Positive*^‡^		*Negative*		*p*-value
	n	(%)	n	(%)	n	(%)		n	(%)	n	(%)	
**Sex**												
Male	313	(55.6)	102	(32.6)	211	(67.4)	< .001	64	(20.4)	249	(79.6)	.108
Female	250	(44.4)	47	(18.8)	203	(81.2)		38	(15.2)	212	(84.8)	
**Age**												
13–14	147	(26.1)	16	(10.9)	131	(89.1)	< .001	10	(6.8)	137	(93.2)	< .001
15–16	217	(38.5)	61	(28.1)	156	(71.9)		51	(23.5)	166	(76.5)	
17–18	199	(35.3)	72	(36.2)	127	(63.8)		41	(20.6)	158	(79.4)	
**Academic grade**												
Secondary	315	(56.0)	65	(20.6)	250	(79.4)	< .001	48	(15.2)	267	(84.8)	.046
Higher secondary	248	(44.0)	84	(33.9)	164	(66.1)		54	(21.8)	194	(78.2)	
**Relationship status**												
Not in relationship	478	(84.9)	114	(23.8)	364	(76.2)	.001	81	(16.9)	397	(83.1)	.087
In a relationship	85	(15.1)	35	(41.2)	50	(58.8)		21	(24.7)	64	(75.3)	
**Socio-economic status (SES)**												
Lower	59	(10.5)	18	(30.5)	41	(69.5)	.413	15	(25.4)	44	(74.6)	.305
Middle	150	(26.6)	34	(22.7)	116	(77.3)		26	(17.3)	124	(82.7)	
Upper	354	(62.9)	97	(27.4)	257	(72.6)		61	(17.2)	293	(82.8)	
**Living with family**												
Yes	488	(86.7)	120	(24.6)	368	(75.4)	.010	87	(17.8)	401	(82.2)	.649
No	75	(13.3)	29	(38.7)	46	(61.3)		15	(20.0)	60	(80.0)	
**Perceived relationship with friends**												
Good	430	(76.4)	98	(22.8)	332	(77.2)	< .001	61	(14.2)	369	(85.8)	< .001
Not good	133	(23.6)	51	(38.3)	82	(61.7)		41	(30.8)	92	(69.2)	
**Regular physical activity**												
Yes	312	(55.4)	80	(25.6)	232	(74.4)	.621	51	(16.3)	261	(83.7)	.224
No	251	(44.6)	69	(27.5)	182	(72.5)		51	(20.3)	200	(79.7)	
**Sleeping satisfaction**												
Yes	469	(83.3)	103	(22.0)	366	(78.0)	< .001	76	(16.2)	393	(83.8)	.008
No	94	(16.7)	46	(48.9)	48	(51.1)		26	(27.7)	68	(72.3)	
**Sleeping status**												
Less than normal	261	(46.4)	75	(28.7)	186	(71.3)	.416	57	(21.8)	204	(78.2)	.097
Normal (7–8 hours)	249	(44.2)	63	(25.3)	186	(74.7)		38	(15.3)	211	(84.7)	
More than normal	53	(9.4)	11	(20.8)	42	(79.2)		7	(13.2)	46	(86.8)	
**Cigarette smoker**												
Yes	64	(11.4)	36	(56.3)	28	(43.8)	< .001	23	(35.9)	41	(64.1)	< .001
No	499	(88.6)	113	(22.6)	386	(77.4)		79	(15.8)	420	(84.2)	
**Internet use**												
Yes	497	(88.3)	140	(28.2)	357	(71.8)	.012	93	(18.7)	404	(81.3)	.314
No	66	(11.7)	9	(13.6)	57	(86.4)		9	(13.6)	57	(86.4)	
**Depression**												
Positive	149	(26.5)	149	(100.0)	0	(0.0)	—	72	(48.3)	77	(51.7)	< .001
Negative	414	(73.5)	0	(0.0)	414	(100.0)		30	(7.2)	384	(92.8)	
**Anxiety**												
Positive	102	(18.1)	72	(70.6)	30	(29.4)	< .001	102	(100.0)	0	(0.0)	—
Negative	461	(81.9)	77	(16.7)	384	(83.3)		0	(0.0)	461	(100.0)	

Note:

^†^Positive depression indicates moderate to severe depression (PHQ-9 ≥ 10)

^‡^Positive anxiety indicates moderate to severe depression (GAD-7 ≥ 10)

The prevalence rates of moderate to severe levels of depression and anxiety were 26.5% and 18.1%, respectively. Based on the PHQ-9 scale, results indicated that minimal, mild, moderate, moderately severe, and severe depression levels were present in 30.0%, 43.5%, 17.2%, 5.9%, and 3.4%, respectively. Based on the GAD-7 scale, results indicated that minimal, mild, moderate, and severe anxiety levels had rates of 43.5%, 38.4%, 13.7%, and 4.4%, respectively. The proportion of moderate to severe depression was higher in (ⅰ) males vs. females, (ⅱ) older participants (17 to 18 years) vs. 13–16 years age group, (ⅲ) those with a higher secondary academic grade vs. secondary grade, (ⅳ) those engaged in a relationship vs. not in relationship, (ⅴ) those living apart from the family vs. those living with family, (ⅵ) those with a suboptimal relationship with friends vs. those with a good relationship, (ⅶ) those with unsatisfactory sleep vs. those with good sleep, (ⅷ) cigarette smokers vs. in non-smokers, (ⅸ) internet users vs. non-users, and (ⅹ) those with anxiety vs. without anxiety. The proportion of anxiety was higher in (ⅰ) older participants (15 to 16 years) vs. the 13–14 years age group, (ⅱ) those with a higher secondary academic grade vs. secondary grade, (ⅲ) those with a suboptimal relationship with friends vs. those with good relationship, (ⅳ) those with unsatisfactory sleep vs. those with good sleep, (ⅴ) cigarette smokers vs. non-smokers, (ⅳ) those with depression vs. those without depression (Table 1).

The results of univariable and multivariable logistic regression analyses are presented in Table 2. Based on unadjusted estimates, depression and anxiety were significantly associated with being older, having higher secondary grades, not having a good perceived relationship with friends, having unsatisfactory sleep, and being a cigarette smoker.

**Table 2:** Regression analyses of factors associated with depression and anxiety among adolescents.

Variables	Depression						Anxiety					
	COR	(95% CI)	*p-*value	AOR	(95% CI)	*p-*value	COR	(95% CI)	*p-*value	AOR	(95% CI)	*p-*value
**Sex (ref. Female)**												
Male	2.09	(1.41–3.10)	< .001	1.35	(0.8–2.27)	.260	1.43	(0.92–2.23)	.109	—	—	—
**Age (ref. 13–14 years)**												
15–16 years	3.20	(1.76–5.82)	< .001	1.82	(0.87–3.78)	.110	4.21	(2.06–8.60)	< .001	2.66	(1.18–6.00)	.018
17–18 years	4.64	(2.56–8.41)	< .001	2.61	(0.96–7.13)	.061	3.56	(1.72–7.36)	.001	1.26	(0.42–3.81)	.680
**Academic Grade (ref. Secondary)**												
Higher secondary	1.97	(1.35–2.88)	< .001	0.91	(0.43–1.92)	.809	1.55	(1.01–2.38)	.047	1.31	(0.61–2.80)	.484
**Relationship status (ref. Not in relationship)**												
In a relationship	2.24	(1.38–3.61)	.001	1.59	(0.87–2.91)	.130	1.61	(0.93–2.78)	.089	—	—	—
**Socio-economic status (SES) (ref. Lower)**												
Middle	0.67	(0.34–1.31)	.239	—	—	—	0.62	(0.30–1.27)	.187	—	—	—
Upper	0.86	(0.47–1.57)	.622				0.61	(0.32–1.17)	.136			
**Living with family (ref. Yes)**												
No	1.93	(1.16–3.21)	.011	1.61	(0.85–3.07)	.147	1.15	(0.63–2.12)	.650	—	—	—
**Perceived relationship with friends (ref. Good)**												
Not good	2.11	(1.39–3.19)	< .001	1.51	(0.91–2.52)	.111	2.70	(1.71–4.26)	< .001	2.10	(1.24–3.56)	.006
**Regular physical activity (ref. Yes)**												
No	1.10	(0.76–1.60)	.621	—	—	—	1.31	(0.85–2.01)	.225	—	—	—
**Sleeping satisfaction (ref. Yes)**												
No	3.41	(2.15–5.39)	< .001	3.17	(1.81–5.53)	< .001	1.98	(1.18–3.31)	.009	0.94	(0.51–1.73)	.833
**Sleeping status (ref. More than normal)**												
Less than normal	1.54	(0.75–3.15)	.237	—	—	—	1.84	(0.79–4.29)	.160			
Normal (7–8 hours)	1.29	(0.63–2.66)	.486				1.18	(0.50–2.82)	.703			
**Cigarette smoker (ref. No)**												
Yes	4.39	(2.57–7.51)	< .001	2.00	(1.01–3.97)	.048	2.98	(1.70–5.24)	< .001	1.67	(0.84–3.33)	.142
**Internet use (ref. No)**												
Yes	2.48	(1.20–5.15)	.015	1.58	(0.68–3.67)	.284	1.46	(0.70–3.05)	.317	—	—	—
**Depression (ref. Negative)**												
Positive^†^	—	—	—	—	—	—	11.97	(7.32–19.56)	< .001	10.22	(6.01–17.38)	< .001
**Anxiety (ref. Negative)**												
Positive^‡^	11.97	(7.32–19.56)	< .001	10.47	(6.11–17.95)	< .001	—	—	—	—	—	—

^\^ *Note*: COR: Crude Odds Ratio; CI: Confidence Interval; AOR: Adjusted Odds Ratio

^†^Positive depression indicates moderate to severe depression (PHQ-9 ≥ 10)

^‡^Positive anxiety indicates moderate to severe depression (GAD-7 ≥ 10)

Additionally, depression was significantly correlated with being male, being engaged in a relationship, not living with the family and being an internet user. Associations for these additional variables were directionally similar for anxiety, but of lower effect size and not statistically significant. Anxiety and depression were strongly associated with each other (Table 2).

Multivariable analyses with all significant factors from univariable analyses entered together in the model revealed that most associated factors lost significance. The participants with unsatisfactory sleep were 3.17 times more likely to be depressed than those who had not (AOR = 3.17; 95% CI = 1.81–5.53, *p* < .001). Likewise, associated factors of depression including cigarette smoking (AOR = 2.00; 95% CI = 1.01–3.97, *p* = .048), and anxiety (AOR = 10.47; 95% CI = 6.11–17.95, *p* < .001), whilst for anxiety, only associations with being 15–16 years (AOR = 2.66; 95% CI = 1.18–6.00, *p* = .018), not having a good perceived relationship with friends (AOR = 2.10; 95% CI = 1.24–3.56, *p* = .006) and depression (AOR = 10.22; 95% CI = 6.01–17.38, *p* < .001) were retained in multivariable analyses (Table 2).

## Discussion

As adolescents carry the future of the nation, mental health is significant for their physical, cognitive, and academic progress. Mental ill-health in adolescents is a significant public health issue that deserves greater levels of attention of researchers. Therefore, the current study examined rates of depression and anxiety as well as potential associated factors in school going adolescents in Dhaka City, Bangladesh.

### Rates of depression and anxiety

In the present study, the findings indicated that nearly one-third of respondents (26.5%) experienced moderate to severe depression. Compared to previous studies in Bangladesh among adolescents, the prevalence of depression in the present study was higher (26.5% vs. 25%) than a previous study conducted in 2013 among 898 adolescents aged 13–16 years in Dhaka City [28]. The prevalence of depression in the present study was lower (26.5% vs. 36.6%) than a study conducted in 2018 among 311 adolescents aged 13–17 years in Dhaka (urban and semi-urban areas) [11] and also lower (26.5% vs. 49%) compared to a previous study conducted in 2012 among 165 adolescents aged 15–19 years selected from two urban schools in Bangladesh [29]. In the present study, one-fifth of respondents (18.1%) experienced moderate to severe anxiety. Direct comparison of this finding is difficult due to a lack of similar Bangladeshi studies.

Table 3 demonstrates the prevalence rates of depression and anxiety among adolescents in Asian jurisdictions. The rate of depression and anxiety across all these studies likely varies in relation to a number of factors. First, given that depression and anxiety represent a spectrum of severity, differences in cut-offs occasioned by different instruments likely will yield differences in prevalence estimates. Second, given strong contextual effects in common mental disorders, rates will vary also across differences in socio-demographic settings, age groups, and age-period-cohort strata.

**Table 3:** Prevalence rates of depression and anxiety among adolescents in Asian jurisdictions.

References	Countries	Depression	Anxiety
[6]	India	25.8%	—
[3]	Pakistan	17.2%	21.4%
[7]	Malaysia	26.2%	—
[8]	Sri Lanka	36%	28%
[30]	Nepal	17%	10%
[2]	Jordan	—	16.3%

### Associations with depression and anxiety

Significant associated factors of depression were having unsatisfactory sleep, being a cigarette smoker, and having anxiety (see Table 2). The findings revealed that participants with unsatisfactory sleep were associated with depression which was similar with previous reports in adolescent populations in Bangladesh [11] and China [31]. It is also consistent with a large-scale study in Norway [32]. This finding also supports a recent case-control study conducted with adolescents [33]. The present report also revealed that adolescents who were currently smoking had greater odds of depression compared to those who did not smoke. This finding is in line with previous reports [34–35] and also consistent with a randomized controlled trial [36]. However, the present study found no statistically significant associations between depression among adolescent students and father’s education and occupation, mother’s education and occupation, living with parents, family size, and school grade.

The present study’s findings also demonstrated that significant factors of anxiety were being older age (15–16 years), not having a good perceived relationship with friends, and having depression. The present report reflected that older adolescents were more prone to being anxious which is in line with a presenting study conducted in Jordan [2]. The present findings revealed that not having a good perceived relationship with friends had greater odds of anxiety compared to those who had a perceived good relationship with friends. This is concurrent with a prior report which found that the perceived care by friends mediated the effect of making friends on social anxiety [37]. The same study concluded that caring relationships with friends and parents each play a role in protecting early adolescents against increasing levels of social anxiety over time. Another report also found that anxiety was associated with self-reported impaired friendship quality [38].

The present study revealed a strong association between anxiety and depression. The assessment of anxiety in the context of depression, and vice versa, is important, given frequent comorbidity with depression and the resulting diagnostic and clinical complications [39]. Anxiety and depression are closely related nevertheless may represent separate domains [40, 41], particularly in adolescents [42]. As expected, anxiety was highly comorbid with depression, such that the anxiety comorbidity rate was 76.5% for depression and the depression comorbidity rate was 41.1% for anxiety. Anxiety comorbidity in depression, and vice versa was also previously found in Bangladesh among different populations [43–45]. This is relevant, as comorbidity negatively affects severity and prognosis [46–48] and early anxiety may increase the probability of occurring depressive disorder [49]. Therefore, from a public health perspective, it is important to focus on both anxiety and depression in combination.

A notable difference was the association between depression and male sex obtained in the univariable analysis. While unusual, such differences have been reported previously in cross-sectional studies [12, 50], and should be interpreted as a function of the local mix of socioeconomic and cultural factors impacting differentially on depression in men and women [50]. Indeed, in the multivariable analysis, male sex was no longer associated with depression, suggesting the sex difference was reducible to other factors that may be specific to Bangladeshi culture in terms of differential impact on sex.

Other factors that were not examined but which may have contributed to the relatively high prevalence rates and sex difference include ethnicity, religion, parental education level, type of housing, alcohol intake, and mental health status of parents [2, 6, 7].

### Limitations

The present study should be interpreted in the light of several limitations. Firstly, the study used self-report methodology that may have prejudiced the outcomes through well-known factors such as memory recall dependence and social desirability. Secondly, the study was cross-sectional in nature which hampers causal attribution. Carrying out a longitudinal study would overcome this limitation in understanding potential causal relationships. Thirdly, the modestly sized sample is only representative of selected schools (using convenient sampling) of Dhaka City in Bangladesh and might not generalize to other Bangladeshi schools or schools in other countries. Therefore, studies employing larger samples from more representative school populations are required. Moreover, the present study used psychometric scales that were previously used/ validated among the general population in Bangladesh rather than adolescent samples. Likewise, the cut-offs of the screening tools were not well established for Bangladeshi adolescents against gold-standard assessments. The exclusion of adolescents who might have dropped out of school also limited the study findings. Finally, the factor ‘sleep’ was included as an associated factor but also may represent a symptom of anxiety and depression. Nevertheless, sleep was included separately because it is a transdiagnostic factor with major functional consequences but also can occur in isolation representing an important associated factor for a range of mental disorders. In other words, it can be both parts of the disorder and a symptom of the disorder.

## Conclusions

The present study gathered baseline data in order to address a confirmed knowledge gap regarding depression and anxiety among adolescents in Bangladesh. The study found a relatively high prevalence of depression and anxiety compared to existing global studies and highlighted a number of associated factors that were associated with depression and/or anxiety. These associated factors, if replicated in longitudinal research, may be targeted by Bangladeshi authorities making policy-level decisions regarding evaluation, prevention, and intervention of mental health conditions among adolescents in Bangladesh.

## Supporting information

S1 File Data set- prevalence of depression, anxiety and associated factors among adolescents in Bangladesh: Findings from a cross-sectional study. (XLSX)

S2 File Questionnaire. (DOCX)
