# Supplementary material for: Prevalence of depression, anxiety and associated factors among school going adolescents in Bangladesh: Findings from a cross-sectional study
Source: PLoS One. 2021 Apr 1;16(4):e0247898. doi: 10.1371/journal.pone.0247898 (PMC8016317; doi:10.1371/journal.pone.0247898)
Supplement: S2 File — (DOCX) [file pone.0247898.s002.docx]

### ****Research Questionnaire (Bangla Version)****

**Section A: Socio-demgraphic Information**

| **ক্রমিক নং** | **প্রশ্ন** | **উত্তর / কোডিং ক্যাটেগরি** |
| --- | --- | --- |
| A_1 | তোমার বয়স কত ? *(বছরে উল্লেখ করবে)* |  |
| A_2 | তোমার লিঙ্গ **চিহ্নিত কর** | ১ = ছেলে ২ = মেয়ে |
| A_3 | তুমি কোন শ্রেণীতে পড়ছো ? | ১ = অষ্টম ২ = নবম  ৩ = দশম ৪ = একাদশ  ৫ = দ্বাদশ |
| A_4 | তোমার পরিবারের মাসিক আয় কত ? (টাকায় লেখ) |  |
| A_5 | তোমার বৈবাহিক অবস্থা | ১= অবিবাহিত  ২ = সম্পর্ক বিদ্যমান (প্রেম) |
| A_6 | তুমি কি তোমার পরিবারের সাথে থাক? | ১= হ্যাঁ  ২= না |
| A_7 | সহপাঠীর সাথে তোমার সম্পর্ক কেমন? | ১= ভালো  ২= ভালো না |

**Section B: Lifestyle-related variables**

| **ক্রমিক নং** | **প্রশ্ন** | **উত্তর / কোডিং ক্যাটেগরি** |
| --- | --- | --- |
| **B_1** | তোমার কি নিয়মিত শারীরিক পরিশ্রম করার অভ্যাস আছে ? | ১ = হ্যাঁ  ২ = না |
| **B_2** | গত ৭ দিনে, তুমি কত ঘন্টা ঘুমিইয়েছো (রাতে) ? |  |
| **B_3** | গত ৭ দিনে, তোমার ঘুম কেমন হয়েছে বলে মনে করেন? | ১ = ভালো  ২= খারাপ |
| **B_4** | তুমি কি বর্তমানে ধূমপান কর? | ১ = হ্যাঁ  ২= না |
| **B_5** | তুমি কি ইন্টারনেট ব্যবহার কর? | ১ = হ্যাঁ  ২= না |

**Section C: Mental Health Assessment**

| **ক্রমিক নং** | **প্রশ্ন** | **উত্তর / কোডিং ক্যাটেগরি** |
| --- | --- | --- |
| **গত ১৪ দিনে নিন্মলিখিত ঘটনাগুলো তোমার সাথে কতবার ঘটেছে বা তুমি কতবার তা অনুভব করেছো ?** | | |
| C_1.1 | কোন কারণে বিচলিত কিংবা উদ্বিগ্ন অনুভব করা | ০ = একদমই না  ১ = মাঝেমাঝে  ২ = অর্ধেকেরও বেশীদিন  ৩ = প্ৰায় প্রতিদিন |
| C_1.2 | ঘুমাতে না পারা বা দুশ্চিন্তা দূর করতে না পারা | ০ = একদমই না  ১ = মাঝেমাঝে  ২ = অর্ধেকেরও বেশীদিন  ৩ = প্ৰায় প্রতিদিন |
| C_1.3 | বিভিন্ন বিষয়ে মাত্রাতিরিক্ত চিন্তা করা | ০ = একদমই না  ১ = মাঝেমাঝে  ২ = অর্ধেকেরও বেশীদিন  ৩ = প্ৰায় প্রতিদিন |
| C_1.4 | বিশ্রাম নিতে অসুবিধা হওয়া (অস্থিরতা) | ০ = একদমই না  ১ = মাঝেমাঝে  ২ = অর্ধেকেরও বেশীদিন  ৩ = প্ৰায় প্রতিদিন |
| C_1.5 | এতাে অস্থির লাগছে যে আপনি কোথাও শান্তভাবে বসে থাকতে পারছিলে না | ০ = একদমই না  ১ = মাঝেমাঝে  ২ = অর্ধেকেরও বেশীদিন  ৩ = প্ৰায় প্রতিদিন |
| C_1.6 | সহজেই বিরক্ত রাগান্বিত হয়ে যাওয়া (খিটখিটে মেজাজ) | ০ = একদমই না  ১ = মাঝেমাঝে  ২ = অর্ধেকেরও বেশীদিন  ৩ = প্ৰায় প্রতিদিন |
| C_1.7 | সবসময় ভয়ে থাকা যেন খারাপ কিছু ঘটতে যাচ্ছে | ০ = একদমই না  ১ = মাঝেমাঝে  ২ = অর্ধেকেরও বেশীদিন  ৩ = প্ৰায় প্রতিদিন |
| **গত ১৪ দিনে নিন্মলিখিত ঘটনাগুলো তোমার সাথে কতবার ঘটেছে বা তুমি কতবার তা অনুভব করেছো?** | | |
| C_2.1 | কাজ করতে অল্প আগ্রহ বা আনন্দ না পাওয়া | ০ = একদমই না  ১ = মাঝেমাঝে  ২ = অর্ধেকেরও বেশীদিন  ৩ = প্ৰায় প্রতিদিন |
| C_2.2 | সব কিছুতেই মন খারাপ, আশাহীন বা বিষন্ন লাগত | ০ = একদমই না  ১ = মাঝেমাঝে  ২ = অর্ধেকেরও বেশীদিন  ৩ = প্ৰায় প্রতিদিন |
| C_2.3 | ঘুমাতে অসুবিধা হত বা খুব বেশি ঘুমা লাগা | ০ = একদমই না  ১ = মাঝেমাঝে  ২ = অর্ধেকেরও বেশীদিন  ৩ = প্ৰায় প্রতিদিন |
| C_2.4 | ক্লান্ত লাগত বা কাজে কম শক্তি বা মনোযোগ আসত না | ০ = একদমই না  ১ = মাঝেমাঝে  ২ = অর্ধেকেরও বেশীদিন  ৩ = প্ৰায় প্রতিদিন |
| C_2.5 | খাবার খেতে ইচ্ছে করত না বা অনেক বেশি খেয়ে ফেলতে | ০ = একদমই না  ১ = মাঝেমাঝে  ২ = অর্ধেকেরও বেশীদিন  ৩ = প্ৰায় প্রতিদিন |
| C_2.6 | নিজেকে ব্যর্থ মনে হত বা নিজেকে পরিবার বা সমাজের কাছে বোঝা মোণে হত | ০ = একদমই না  ১ = মাঝেমাঝে  ২ = অর্ধেকেরও বেশীদিন  ৩ = প্ৰায় প্রতিদিন |
| C_2.7 | কোন কিছুতে মনযােগ দিতে সমস্যা হত যেমন পড়াশুনা করতে বসা | ০ = একদমই না  ১ = মাঝেমাঝে  ২ = অর্ধেকেরও বেশীদিন  ৩ = প্ৰায় প্রতিদিন |
| C_2.8 | এত আস্তে চলাফেরা করতে বা কথা বলতে রা অন্য লোকেরা সেটা লক্ষ্য করে বা এঁকবারে উল্টোটা – এতটা চঞ্চল যে সাধারণ মানুষের চেয়ে বেশি চলা ফেরা করতে | ০ = একদমই না  ১ = মাঝেমাঝে  ২ = অর্ধেকেরও বেশীদিন  ৩ = প্ৰায় প্রতিদিন |
| C_2.9 | মরে গেলই ভালো হত বা নিজেকে নিজে আঘাত করে শেষ করতে  ইচ্ছা হত | ০ = একদমই না  ১ = মাঝেমাঝে  ২ = অর্ধেকেরও বেশীদিন  ৩ = প্ৰায় প্রতিদিন |

### Research Questionnaire (English Version)

**Section A: Socio-demographic information**

| **SI No** | **Questions** | **Answers/ coding** | |
| --- | --- | --- | --- |
| A_1 | Age (years): | | |
| A_2 | Sex | | 1. Male 2. Female |
| A_3 | Academic level | | 1. Class 6 2. Class 7  3. Class 8 4. Class 9  5. Class 10 6. Class 11  7. Class 12 |
| A_4 | Monthly family income (BDT) | |  |
| A_5 | Relationship status | | 1. Single  2. In a relation |
| A_6 | Living with whom | | 1. With family  2. Apart from family |
| A_7 | Relationship with your classmates | | 1. Good  2. Not good |

**Section B: Lifestyle-related factors**

| **SI No** | **Questions** | **Answers/ coding** |
| --- | --- | --- |
| B_1 | Do you perform any physical exercise daily? | 1. Yes 2. No |
| B_2 | On average, how long did you sleep during the last 7 days? |  |
| B_3 | How would you rate your sleep quality overall during the last 7 days? | 1. Satisfied  2. Not satisfied |
| B_4 | Do you smoke cigarette currently? | 1. Yes 2. No |
| B_5 | Do you use internet? | 1. Yes  2. No |

**Section C: Mental Health Assessment**

| **SI No** | **Questions** | **Answers/ coding** |
| --- | --- | --- |
| **Over the last 2 weeks, how often have you been bothered by the following problems?** | | |
| C_1.1 | Feeling nervous, anxious or on edge | 0 = Not at all  1 = Several days  2 = Over half the days  3 = Nearly every day |
| C_1.2 | Not being able to stop or control worrying | 0 = Not at all  1 = Several days  2 = Over half the days  3 = Nearly every day |
| C_1.3 | Worrying too much about different things | 0 = Not at all  1 = Several days  2 = Over half the days  3 = Nearly every day |
| C_1.4 | Trouble relaxing | 0 = Not at all  1 = Several days  2 = Over half the days  3 = Nearly every day |
| C_1.5 | Being so restless that it is hard to sit still | 0 = Not at all  1 = Several days  2 = Over half the days  3 = Nearly every day |
| C_1.6 | Becoming easily annoyed or irritable | 0 = Not at all  1 = Several days  2 = Over half the days  3 = Nearly every day |
| C_1.7 | Feeling afraid as if something awful might happen | 0 = Not at all  1 = Several days  2 = Over half the days  3 = Nearly every day |
| **Over the last 2 weeks, how often have you been bothered by the following problems?** | | |
| C_2.1 | Little interest or pleasure in doing things | 0 = Not at all  1 = Several days  2 = Over half the days  3 = Nearly every day |
| C_2.2 | Feeling down, depressed, or hopeless | 0 = Not at all  1 = Several days  2 = Over half the days  3 = Nearly every day |
| C_2.3 | Trouble falling or staying asleep, or sleeping too much | 0 = Not at all  1 = Several days  2 = Over half the days  3 = Nearly every day |
| C_2.4 | Feeling tired or having little energy | 0 = Not at all  1 = Several days  2 = Over half the days  3 = Nearly every day |
| C_2.5 | Poor appetite or overeating | 0 = Not at all  1 = Several days  2 = Over half the days  3 = Nearly every day |
| C_2.6 | Feeling bad about yourself — or that you are a failure or have let yourself or your family down | 0 = Not at all  1 = Several days  2 = Over half the days  3 = Nearly every day |
| C_2.7 | Trouble concentrating on things, such as reading the newspaper or watching television | 0 = Not at all  1 = Several days  2 = Over half the days  3 = Nearly every day |
| C_2.8 | Moving or speaking so slowly that other people could have noticed? Or the opposite — being so fidgety or restless that you have been moving around a lot more than usual | 0 = Not at all  1 = Several days  2 = Over half the days  3 = Nearly every day |
| C_2.9 | Thoughts that you would be better off dead or of hurting yourself in some way | 0 = Not at all  1 = Several days  2 = Over half the days  3 = Nearly every day |
